# A protocol for automated a posteriori adaptive meshing with SimVascular: a test case

**DOI:** 10.1186/s13104-020-05057-7

**Published:** 2020-04-22

**Authors:** Akash Gupta, Ethan Kung

**Affiliations:** 1 Department of Mechanical Engineering, Clemson University, Fluor Daniel Building, Clemson, SC 29634 USA; 2 Department of Mechanical Engineering and Bioengineering, Clemson University, Fluor Daniel Building, Clemson, SC 29634 USA

**Keywords:** Adaptive meshing, Iterative protocol, A posteriori error indicators, SimVascular, Computational fluid dynamics

## Abstract

**Objective:**

Operational details regarding the use of the adaptive meshing (AM) algorithm available in the SimVascular package are scarce despite its application in several studies. Lacking these details, novice users of the AM algorithm may experience undesirable outcomes post-adaptation such as increases in mesh error metrics, unpredictable increases in mesh size, and losses in geometric fidelity. Here we present a test case using our proposed iterative protocol that will help prevent these undesirable outcomes and enhance the utility of the AM algorithm. We present three trials (conservative, moderate, and aggressive settings) applied to a scenario modelling a Fontan junction with a patient-specific geometry and physiologically realistic boundary conditions.

**Results:**

In all three trials, an overall reduction in mesh error metrics is observed (range 47%–86%). The increase in the number of elements through each adaptation never exceeded the mesh size of the pre-adaptation mesh by one order of magnitude. In all three trials, the protocol resulted in consistent, repeatable improvements in mesh error metrics, no losses of geometric fidelity and steady increments in the number of elements in the mesh. Our proposed protocol prevented the aforementioned undesirable outcomes and can potentially save new users considerable effort and computing resources.

## Introduction

The finite element (FE) method is a powerful tool for simulating complex haemodynamics observed in cardiovascular flows. A necessity for FE simulations is a converged mesh, i.e., the domain of interest discretized to a sufficient resolution to capture the internal flow physics. Adaptive meshing(AM) algorithms have been shown to be capable of producing converged meshes whose computational cost is orders of magnitude smaller than a uniform mesh of equivalent quality [1], thus reducing computational cost while maintaining accuracy.

The AM algorithm [2] available within the open-source SimVascular package [3] has been utilized in several previous studies [4–8] and employs a posteriori error indicators, specifically, the Interpolation Error. However, details regarding AM algorithm parameter description are lacking in previous literature, and operational guidelines for AM approaches are similarly scarce. In addition, SimVascular’s implementation of this algorithm provides Mean Interpolation Error (MIE) as an output, but MIE is not defined in Ref [2], nor in the SimVascular documentation. Lacking operational guidelines, our initial attempts at utilizing the algorithm produced undesirable outcomes such as:

- Adapted meshes with fewer elements than the initial mesh, whose quality is worse than the initial mesh, i.e., increased mesh error metric post-adaptation with mesh coarsening.
- Adapted meshes with a greater number of elements than the initial mesh, but of worse quality, i.e., increased mesh error metric post-adaptation with mesh refinement.
- An increase in mesh size post-adaptation greater than that of the initial mesh by an order of magnitude.
- Deterioration of the geometric fidelity of adapted meshes when compared to the original geometry.

In this study, we present an example application of a proposed AM protocol, whose objective is to help prevent occurrences of the aforementioned undesirable outcomes, and produce consistent, repeatable improvement in the mesh error metric when using the AM algorithm available in the SimVascular software package.

## Main text

### Methods

#### Description of the proposed protocol

Our proposed protocol utilizes the AM algorithm [2] included in SimVascular [3], an open source software package. The output of this protocol is several adapted meshes and the corresponding FE simulation solutions. Since the overall goal of FE simulations is to achieve sufficient resolution for the parameters of interest (PoI), which are quantities derived from the simulated flow physics important to the research question at hand, the user selects the adapted mesh which adequately resolves the PoI with a minimum number of elements. The protocol consists of an iterative procedure with several operational recommendations. This iterative procedure performs serial adaptations of an initial input mesh using SimVascular’s AM algorithm. In each iteration, an adapted mesh is produced which is then supplied to the subsequent iteration as the input mesh. In each iteration, the input parameters to the AM algorithm are gradually refined to ensure progressive improvements in the mesh. The operational recommendations are a set of suggestions regarding the initial mesh and the settings prescribed to the iterative procedure that can help prevent the occurrence of the aforementioned undesirable outcomes.

#### The iterative procedure

**Fig. 1:**
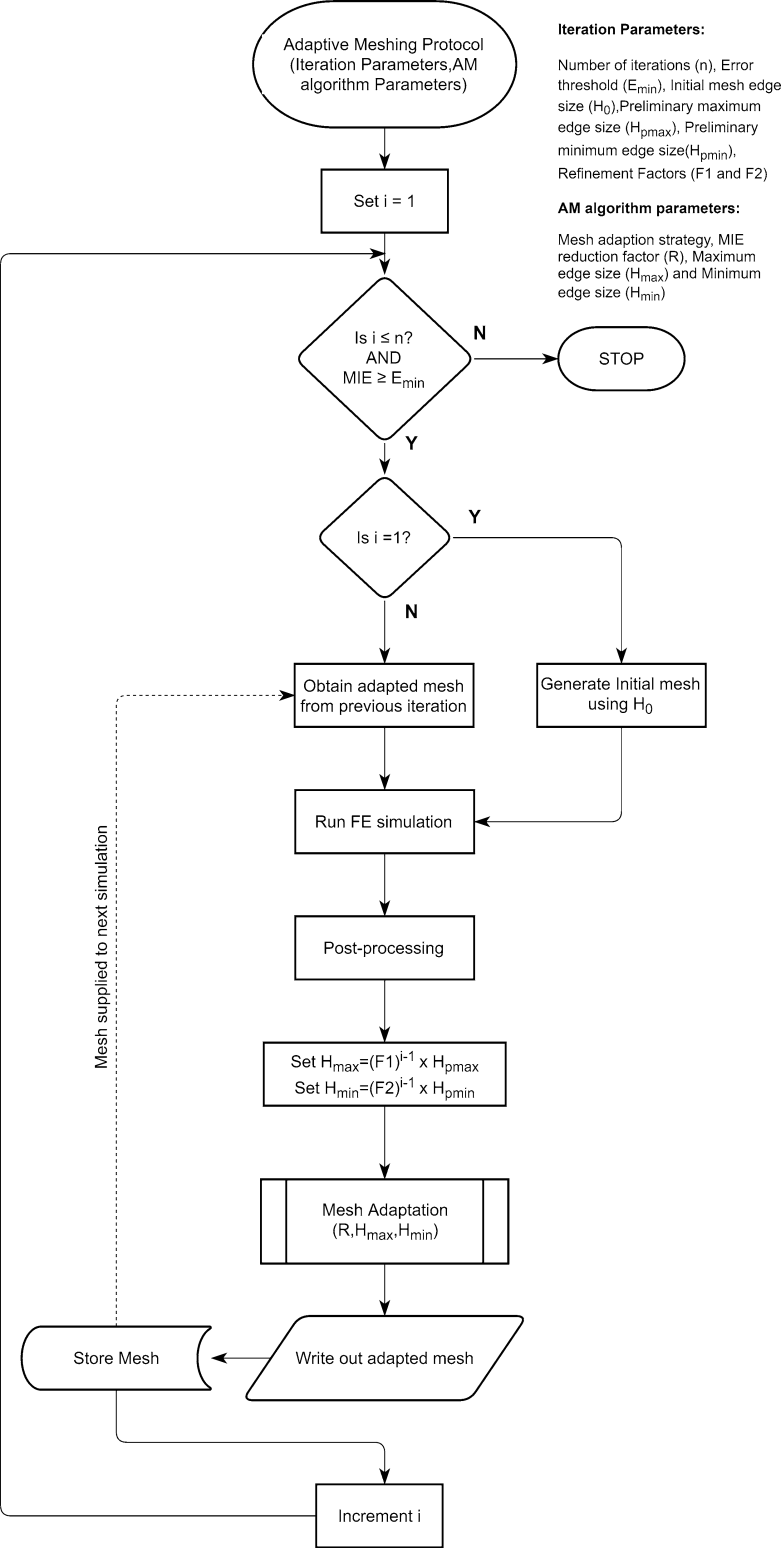
Flow diagram detailing the logic of the proposed AM protocol. Diagram is constructed according to the ISO 5807:1985 standard

The iterative procedure (Fig. 1) is governed by parameters prescribed to it, which may be broadly divided into two categories:

1. Iteration parameters: Number of iterations (n), Error threshold (E_min_), Initial mesh edge size (H_0_), Refinement Factors (F1 and F2), Preliminary maximum edge size (H_pmax_), Preliminary minimum edge size (H_pmin_).
2. AM algorithm parameters:
  - Mesh adaptation strategy: Isotropic versus anisotropic. Our protocol exclusively uses the anisotropic setting as recommended by the authors of the AM algorithm [2].
  - MIE reduction factor (R): The AM algorithm attempts to reduce the MIE through each iteration by this factor. It should always be less than 1.
  - Maximum edge size (H_max_) and Minimum edge size (H_min_): The maximum and minimum edge size parameters constrain the size of the elements produced in the adapted mesh.

The iterative procedure begins with a uniform mesh where the global edge size for the bulk volume of the geometry is set to H_0_. In order to refine the mesh progressively, the protocol reduces H_min_ and H_max_ by refinement factors F1 and F2, respectively, in each iteration. For the first iteration, H_max_ and H_min_ are set equal to H_pmax_ and H_pmin_, respectively. The error threshold, E_min_, is the user-defined value of MIE at which the protocol will stop operation irrespective of the number of completed iterations. Since the MIE of the initial mesh may be unknown when the protocol is initiated, the initial value of E_min_ should be set to an arbitrarily large value. Once the MIE of the initial mesh is determined after the post-processing step of the first iteration, E_min_ is updated to the desired value, i.e., a certain proportion (less than 1) of the MIE of the initial mesh.

#### Operational recommendations

In addition to the iterative procedure described above, the following operational recommendations are critical for the proper working of the protocol:

1. The value of H_max_ prescribed to the AM algorithm should always be lesser than H_0_, to prevent mesh coarsening.
2. We have observed that the AM algorithm does not affect faces to which a boundary layer mesh is prescribed. It is our suggestion that this behavior is utilized to preserve the geometric fidelity of “wall” faces and other complex faces by prescribing a fine face mesh and a boundary layer mesh. The fine face mesh and boundary layer mesh prevent changes to those faces, thereby preserving geometric fidelity without significantly increasing the total mesh size.
3. In the initial mesh, the mesh for the gross volume can be quite coarse. The value of H_0_ can be one order of magnitude larger than the edge size prescribed to the wall face.

#### An example application of the protocol

**Fig. 2:**
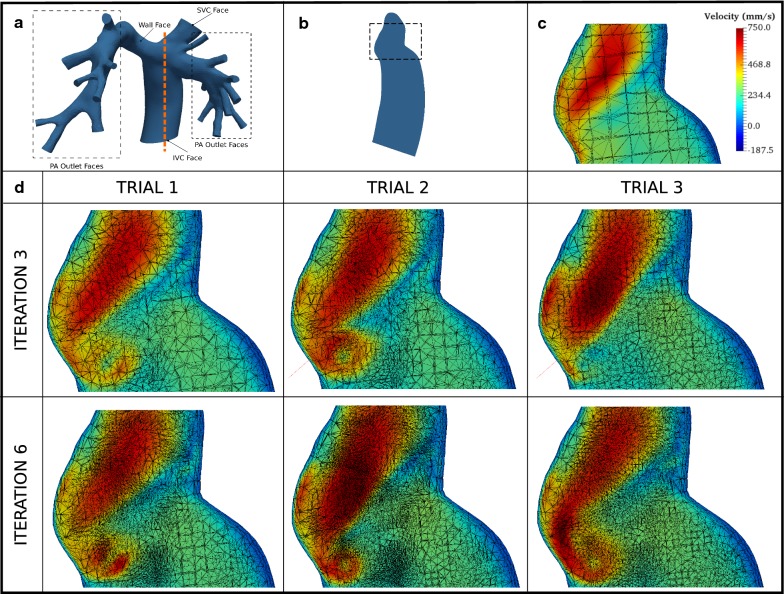
Patient-specific geometry used in all trials and velocity plots overlaid on adapted meshes for all trials at the last timestep corresponding to 1.664 s. **a** Faces at which boundary conditions were applied are marked. Dotted orange line represents location of slice plane; **b** profile of the slice shown in (**a**), dashed box indicates location of detail view; **c** detail view for the initial mesh that is common to all trials, velocity scale shown applies to all detail views; **d** detail views with velocity map and adapted mesh for iterations 3 and 6 of all trials

To demonstrate the working of the protocol, we consider a scenario modelling a Fontan surgical junction with physiologic flow and a patient-specific geometry derived from the MRI scan of a patient with a 19 mm-diameter extracardiac conduit (Fig. 2a). The initial mesh is identical in all three trials and was generated using MeshSim (Simmetrix Inc., NY). In the subsequent section, we present three trial runs of the protocol designated Trials 1, 2 and 3, which represent conservative, moderate and aggressive approaches to MIE reduction, respectively. In all trials E_min_ was set to the value of 30. For this example, we consider the PoI to be the pressure developed at the IVC and SVC faces. A detailed description of the physiologically realistic boundary conditions, initial mesh, simulation and protocol settings are provided in Additional File 1.

### Results

**Fig. 3:**
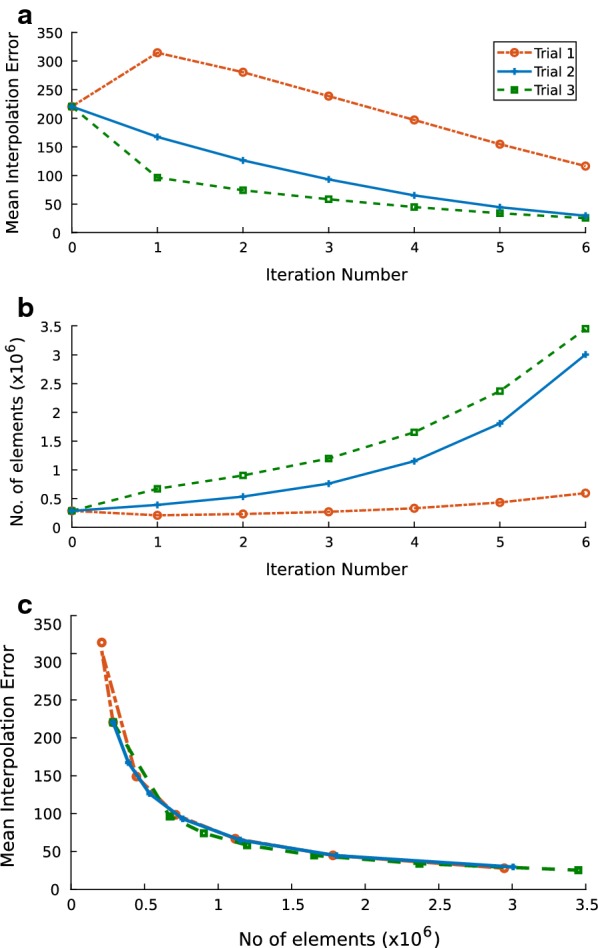
**a** Plot of the MIE versus iteration for all trials; **b** plot of number of elements versus iteration for all trials; **c** Plot of MIE versus the number of elements for all trials

The net reduction in the MIE after six iterations of each trial is 86% for Trials 2 and 3 and 47% for Trial 1. Trials 2 and 3 representing the aggressive and moderate settings exhibit monotonous downward trends (Fig. 3a). Conversely, for Trial 1 an initial uptick is followed by a steady decreasing trend. Trials 2 and 3 are characterized by steady increases in the number of elements while in Trial 1, an initial drop in the mesh size is observed, after which a modest increasing trend is established (Fig. 3b). In all trials, after the first iteration the increase in the number of elements is predictable and never exceeds the mesh size of the pre-adaptation mesh by more than an order of magnitude. In Trials 2 and 3, the number of elements escalates by one order of magnitude over six iterations, where a milder increase occurs over the entirety of Trial 1. When the progression of the MIE is examined vis-à-vis the number of elements in the adapted mesh, all three trials exhibit a decreasing trend for the MIE in a similar fashion (Fig. 3c).

The evolution of the adapted mesh at the region where the IVC and SVC flows collide is shown in Fig. 2d. Starting from the same initial mesh (Fig. 2c), all three trials exhibit the formation of a vortex in approximately the same region by the sixth iteration. In all three trials it can be observed that the mesh density increases in and around the regions where the velocity gradient is large (i.e. in and around the vortex).

### Discussion

The overall objective of our proposed protocol is to produce consistent decreases in the MIE, facilitate predictable increases in the number of elements and prevent losses in geometric fidelity. With the exception of the first iteration of Trial 1, all iterations of all trials exhibit steady reductions in the MIE. These observations are an indication that even with conservative inputs prescribed to the protocol, an overall improvement in the mesh error metrics can be achieved.

All three trials exhibit predictable increases in mesh size through subsequent adaptations, with the exception of the first iteration of Trial 1. A rectification (described in Additional file 1) made to Trial 2 prevented a decrease in the number of elements. It is evident that the user can exercise control over the number of elements in the adapted meshes by modifying the parameters prescribed to the protocol, specifically, H_pmax_, H_pmin_, and the factors F1 and F2, to obtain predictable increases in the number of elements.

All three trials exhibit declining MIE at different rates, and hence with a sufficient number of iterations, the conservative settings can achieve the same reduction in MIE as the aggressive settings. From Fig. 2d it is evident that starting from the same mesh, the location and magnitude of the vortex at the sixth iteration is approximately the same for all three settings we investigated. With these observations in mind, we recommend initiating the protocol with conservative settings. Compared to the aggressive or moderate settings, since the conservative setting reduces H_max_ and H_min_ in smaller steps, the increase in the number of elements (and therefore the computational cost) from one iteration to the next is smaller. This means that the results of the early iterations with conservative settings can be obtained significantly faster than with aggressive settings. The user can monitor the change in the MIE and PoI, and adjust the protocol parameters if necessary, without a substantial time investment.

In a typical FE simulation study, the final choice of the adapted mesh is governed by the resolution of the PoI. The degree of correlation between the MIE and the PoI is dependent upon the phenomenon being investigated; hence it is not necessary that the MIE directly correlate with the error in a PoI. We would recommend that users monitor the change in both the MIE and PoI through every iteration of the protocol. The threshold below which the differences in the PoI can be considered negligible should be determined by the user based on the context of the problem. In a manner similar to a typical mesh independence analysis, the user would choose the adapted mesh that has the smallest number of elements and offers a PoI change from one iteration of the protocol to the next that is within the user defined threshold.

While the proposed protocol was designed to work with the AM algorithm available in SimVascular, the first component of the protocol, i.e., the iterative procedure, could form the basis for iterative procedures valid for other AM algorithms. Iterative procedures are prime candidates for automation and use on supercomputing clusters, potentially reducing the number of man-hours necessary to obtained converged meshes for multiple geometries. With significant advances being made in the field of AM, we hope that our proposed protocol will encourage the publication of similar “lessons learnt” documents for existing and novel AM algorithms, allowing the utilization of their capabilities to the fullest.

## Limitations

- Since this protocol involves the prescription of a preset boundary layer mesh, quantities such as wall shear stress may not be affected by mesh adaptation.
- We have tested this protocol with meshes generated by the commercial MeshSim module. Testing this protocol with the open-source TetGen module could be a promising avenue for future work.
- Here we present an example application of our proposed protocol, and we have not attempted to formally characterize the convergence rate of the algorithm, nor have we explored the question of what degree of geometric complexity (i.e. presence of singularities) justifies the application of this protocol as these are highly dependent on the problem in question.

## Supplementary information

**Additional file 1.** Additional Methods with details of initial mesh generated for all trials, simulation details for example application of the protocol, description of protocol settings and rectification settings for Trial 3. **Table S1.** Protocol parameters prescribed to Trials 1, 2, and 3. **Table S2.** The time required for meshing and simulation for each iteration of all trials. Iteration 0 corresponds to the initial mesh. **Figure S1.** Flow waveforms prescribed to the superior vena cava (SVC) and inferior vena cava (IVC) faces. Plot showing the second cardiac cycle in the simulation where the resulting data was used for the mesh interpolation error calculation in each iteration of all trials.

## Data Availability

Not applicable.

## Abbreviations

AM: Adaptive meshing
FE: Finite element
PoI: Parameter(s) of interest
MIE: Mesh interpolation error
n: Number of iterations
E_min_: Error threshold
H_0_: Initial mesh edge size
H_max_: Maximum edge size
H_min_: Minimum Edge Size
F1: Refinement factor for H_max_
F2: Refinement factor for H_min_
H_pmax_: Preliminary maximum edge size
H_pmin_: Preliminary minimum edge size
R: Mesh interpolation error reduction factor
