# Supplementary material for: A protocol for automated a posteriori adaptive meshing with SimVascular: a test case
Source: BMC Res Notes. 2020 Apr 22;13:229. doi: 10.1186/s13104-020-05057-7 (PMC7178633; doi:10.1186/s13104-020-05057-7)
Supplement: Supplementary file 1 — Additional file 1. Additional Methods with details of initial mesh generated for all trials, simulation details for example application of the protocol, description of protocol settings and rectification settings for Trial 3. Table S1. Protocol parameters prescribed to Trials 1, 2, and 3. Table S2. The time required for meshing and simulation for each iteration of all trials. Iteration 0 corresponds to the initial mesh. Figure S1. Flow waveforms prescribed to the superior vena cava (SVC) and inferior vena cava (IVC) faces. Plot showing the second cardiac cycle in the simulation where the resulting data was used for the mesh interpolation error calculation in each iteration of all trials. [file 13104_2020_5057_MOESM1_ESM.docx]

**ADDITIONAL METHODS:**

**Details of initial mesh generated for all trials:**

The mesh generated for the initial iteration had a mesh edge size of 0.25H_0_ for the inlet and outlet faces, and 0.45H_0_ for the wall face, respectively which were sufficient for maintaining the fidelity of the geometry. Additionally, two layers of boundary layer mesh refinement (each layer has mesh edge size H_0_/8) were prescribed to all of the faces, in accordance with the operational recommendations, to prevent losses in geometric fidelity.

***Simulation details for example application of the protocol***

Physiologically realistic flow waveforms were obtained by scaling the results presented by Kung et al.[1] at 1 metabolic equivalent , such that the cardiac index matched that of Fontan patients reported by Tominaga et al.[2] We prescribed two cardiac cycles of these waveforms to faces corresponding to the Inferior Vena Cava (IVC) and Superior Vena Cava (SVC). Zero pressure boundary conditions were imposed on the pulmonary outlet faces and no-slip boundary condition on the walls. The fluid was modeled to be Newtonian with a density of 1092.4 kg/m^3^ and a dynamic viscosity of 0.0041 Pa·s, similar to that of blood. A time step size of 1 millisecond was used in all simulations of all trials. For each simulation, the MIE was calculated based on the results for the second cardiac cycle, corresponding to 0.832s to 1.664s (Figure S1).

All three trials were conducted on a single node (Intel Xeon C4130, 40 cores, 128GB RAM) of the Clemson Palmetto supercomputing cluster. All simulations were run with 40 parallel threads; all other processes involved in the protocol such as meshing and post-processing were performed as single thread processes.

***Description of protocol settings***

The values of the protocol parameters prescribed to Trials 1,2 and 3 are specified in Table S1. We describe the settings of Trial 3 as aggressive since the values of H_pmax_ and H_pmin_ are one order of magnitude smaller than H_0_. In contrast, H_pmax_ and H_pmin_ in Trial 1 are equal to H_0_, hence regarded to be conservative. In Trial 2, the values of H_pmax_ and H_pmin_ were set to the values of H_max_ and H_min_ of the second iteration of Trial 1, respectively, in addition to a smaller value of F2. These settings cause the H_max_ and H_min_ of Trial 2 in each iteration to be intermediate between the values of H_max_ and H_min_ of Trials 1 and 3. We discuss the rationale behind this choice for Trial 3 in the results and discussion section.

**ADDITIONAL RESULTS AND DISCUSSION:**

***Rectification settings for Trial 2:***

In Trial 1, steady decreases in the MIE occur from the second iteration onward, which indicate that the prescribed values of H_pmax_ and H_pmin_ are too large, causing mesh coarsening, while the values for H_max_ and H_min_ for the second iteration are sufficiently small to prevent mesh coarsening. By setting the values of H_pmax_ and H_pmin_ of Trial 2 to the value of H_max_ and H_min_ of the second iteration of Trial 1, respectively, we ensure that for all iterations of Trial 2, the values of H_max_ and H_min_ are sufficiently refined to prevent the occurrence of an increase in MIE and mesh coarsening.

**REFERENCES:**

1. Kung E, Pennati G, Migliavacca F, Hsia T-Y, Figliola R, Marsden A, Giardini A (2014) A Simulation Protocol for Exercise Physiology in Fontan Patients Using a Closed Loop Lumped-Parameter Model. J Biomech Eng 136:081007

2. Tominaga Y, Kawata H, Iwai S, Yamauchi S, Kugo Y, Hasegawa M, Kayatani F, Takahashi K, Aoki H (2018) Left ventricular function after a Fontan operation in patients with pulmonary atresia with an intact ventricular septum. Interact Cardiovasc Thorac Surg 1–6

**ADDITIONAL TABLES**

Table S1: Protocol parameters prescribed to Trials 1, 2, and 3.

| **Trial No.** | **Initial mesh edge size** | **Preliminary maximum mesh edge size** | **Preliminary minimum mesh edge size** | **Max edge size reduction factor** | **Minimum edge size reduction factor** | **MIE reduction factor** |
| --- | --- | --- | --- | --- | --- | --- |
|  | **H_0_ (mm)** | **H_pmax_ (mm)** | **H_pmin_ (mm)** | **F1** | **F2** | **R** |
| 1 | 2.0 | 2.0 | 2.0 | 0.9 | 0.8 | 0.5 |
| 2 | 2.0 | 1.275 | 0.738 | 0.9 | 0.75 | 0.5 |
| 3 | 2.0 | 0.7 | 0.7 | 0.95 | 0.8 | 0.5 |

Table S2: The time required for meshing and simulation for each iteration of all trials. Iteration 0 corresponds to the initial mesh.

|  | **Trial 1** | | **Trial 2** | | **Trial 3** | |
| --- | --- | --- | --- | --- | --- | --- |
| **Iteration Number** | **Meshing Time (s)** | **Simulation Time (min)** | **Meshing Time (s)** | **Simulation Time (min)** | **Meshing Time (s)** | **Simulation Time (min)** |
| 0 | 22.75 | 57.32 | 25.48 | 61.30 | 23.4 | 55.08 |
| 1 | 3.32 | 45.53 | 9.51 | 85.83 | 8 | 143.65 |
| 2 | 3.08 | 49.13 | 15.61 | 118.12 | 10.9 | 198.33 |
| 3 | 3.86 | 56.92 | 28.59 | 174.50 | 18.11 | 267.33 |
| 4 | 4.42 | 71.55 | 55.31 | 269.67 | 33.15 | 372.33 |
| 5 | 6.64 | 90.23 | 101.13 | 428.33 | 56.03 | 567.33 |
| 6 | 11.67 | 127.43 | 182.76 | 745.00 | 87.22 | 801.83 |
| Total | 55.74 | 498.12 | 418.39 | 1,882.75 | 236.81 | 2,405.90 |

**ADDITIONAL FIGURES:**

**
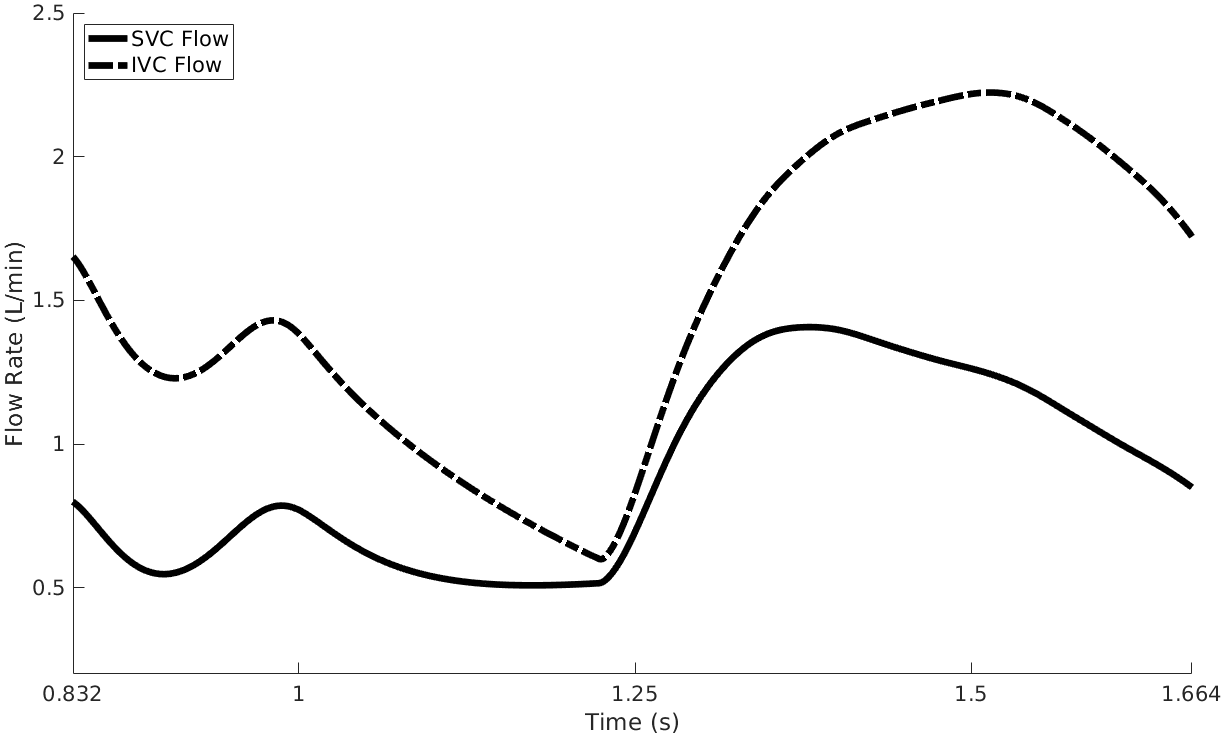
**

**Figure S1:** Flow waveforms prescribed to the superior vena cava (SVC) and inferior vena cava (IVC) faces. Plot showing the second cardiac cycle in the simulation where the resulting data was used for the mesh interpolation error calculation in each iteration of all trials.
